# Recurrent Takotsubo Syndrome Due to Hypoglycemic Attacks

**DOI:** 10.7759/cureus.32527

**Published:** 2022-12-14

**Authors:** Hideya Itagaki, Yoshinobu Abe, Tomoyuki Endo

**Affiliations:** 1 Department of Emergency and Disaster Medicine, Tohoku Medical and Pharmaceutical University Hospital, Sendai, JPN

**Keywords:** coma, systolic blood pressure, heart rate, anorexia nervosa, hypoglycemic attacks, takotsubo syndrome

## Abstract

Takotsubo syndrome (TTS) is a disorder with transient cardiac dysfunction triggered by stress. Rarely, hypoglycemia can also trigger TTS, but there are no case reports of repeated TTS due to hypoglycemia. We report the case of a 51-year-old Japanese woman who was brought to the emergency department with impaired consciousness and shock vitals. Blood tests revealed severe hypoglycemia. She also had an abnormal electrocardiogram with a QS pattern in the anterior thoracic guidance, which led to the diagnosis of TTS after repeated echocardiographic evaluation by a cardiologist. The diagnosis of hypoglycemic coma was made, and the patient was admitted to the intensive care unit (ICU). The patient had anorexia nervosa and had been suffering from a hypoglycemic coma due to anorexia for some time. The patient had a history of hypoglycemic coma about one year before and had been hospitalized in the ICU with TTS at that time. We report the world's first case of repeated TTS due to hypoglycemia. Since hypoglycemia is hemodynamically associated with increased heart rate and systolic blood pressure, TTS should be included in the differential diagnosis when shock vitals are repeated in patients with frequent hypoglycemia.

## Introduction

Takotsubo syndrome (TTS) is transient heart dysfunction with a characteristic ventricular contraction pattern in the absence of overt coronary artery disease [1-6]. It is a rare, recurrent disorder, and no case of hypoglycemia-triggered repeated TTS has been reported. We encountered a patient with anorexia nervosa with a history of hypoglycemia-associated TTS who presented with impaired consciousness and shock vitals, which led to a diagnosis of hypoglycemia-triggered TTS.

## Case presentation

A 51-year-old Japanese woman was brought to the emergency department with a chief complaint of impaired consciousness. She had eaten her usual amount of food, talked with her family, and gone to bed at her usual time until the day before; however, on the morning of the visit, she was found unresponsive in her room. Her height and weight at the time of the visit were 160 cm and 22 kg, respectively. She had received psychiatric treatment for anorexia nervosa for many years and had been prescribed mirtazapine, trazodone, and clonazepam. She had been frequently hospitalized for anorexia-induced hypoglycemic coma. Approximately a year ago, she was hospitalized for hypoglycemic coma, which was complicated by TTS-induced cardiogenic shock, and treated in the intensive care unit (ICU).

**Figure 1:**
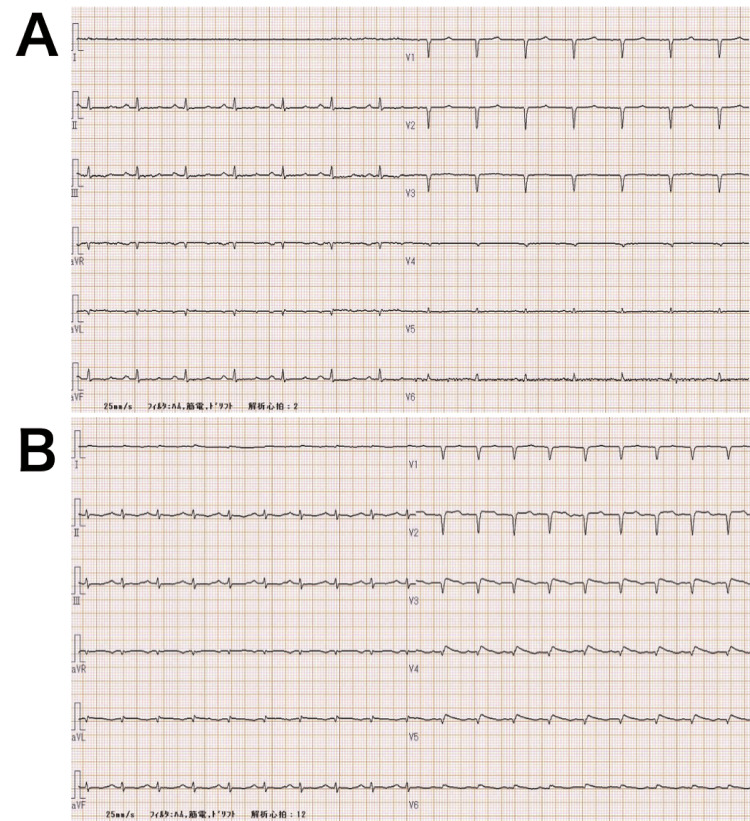
Electrocardiogram on admission (A), electrocardiogram on the second day of admission (B). (A) QS pattern in leads V1–V4. (B) QS pattern in leads V1–V6 leads, with mild ST-segment elevation in leads V2–V6.

**Figure 2:**
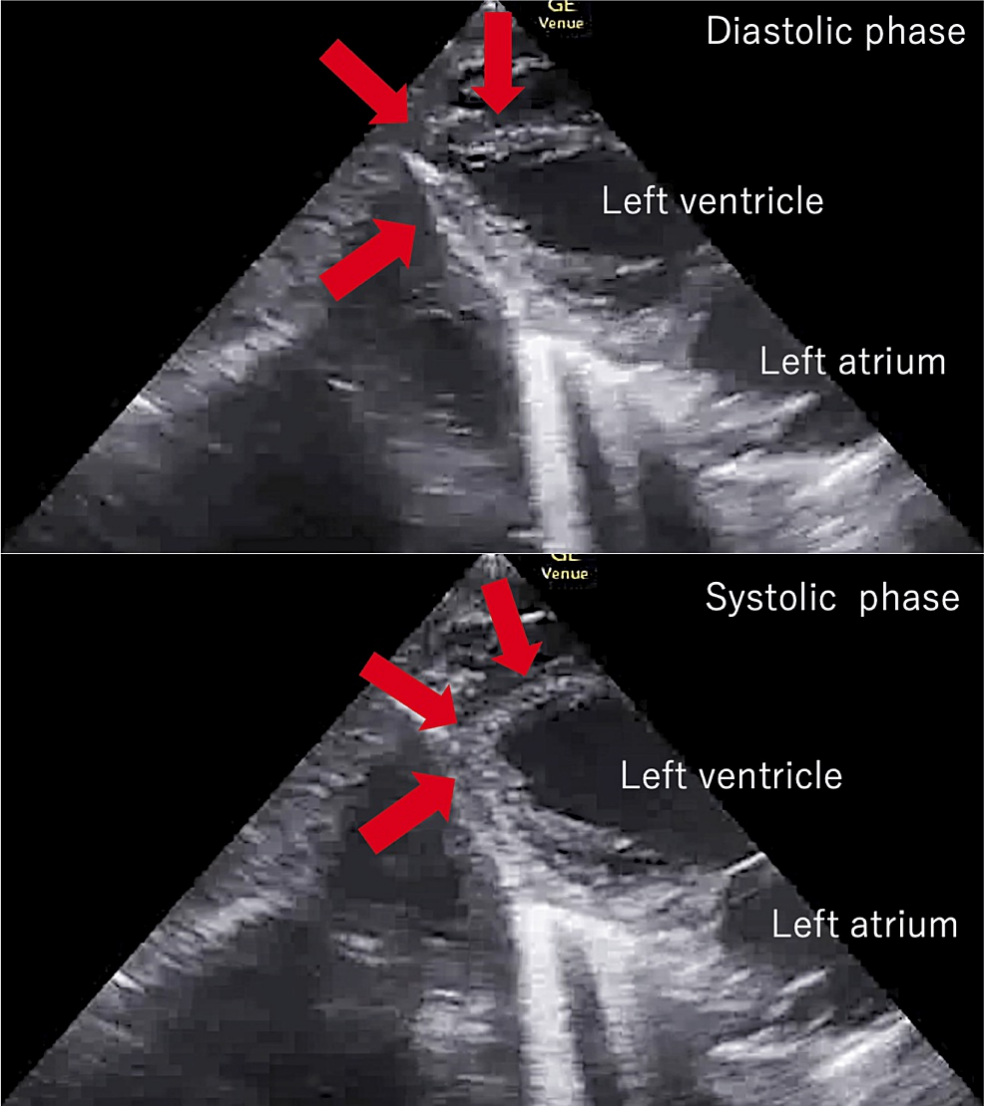
Echocardiogram on admission. Comparison of diastolic and systolic views. Only the apex of the left ventricle (red arrow) contracted.

On arrival, she was unconscious and in shock; her vitals were as follows: Glasgow Coma Scale score, E1V1M1, blood pressure, 85/59 mmHg; pulse, 109 bpm; respiratory rate, 25 breaths/min (similar to mandibular breathing); oxygen saturation, 91% (15 L of oxygen and bag-valve-mask ventilation); and body temperature, 36.0°C. On the third day of hospitalization, the patient responded to voice calls and her awareness improved until she was able to communicate. Blood tests revealed elevated levels of hepatobiliary enzymes (total bilirubin: 3.01 mg/dL, aspartate transaminase: 2893 U/L, alanine transaminase: 1686 U/L, lactate dehydrogenase: 788 U/L, alkaline phosphatase: 335 U/L, gamma-glutamyl transferase: 254 U/L), increased blood urea nitrogen (BUN)/creatinine (Cre) ratio (BUN/Cre: 52 mg/dL/0.73 mg/dL), hyperkalemia (5.8 mmol/L), hyperphosphatemia (6.8 mg/dL), decreased low-density lipoprotein cholesterol and triglycerides (total cholesterol: 151 mg/dL, triglycerides: <5 mg/dL, high-density lipoprotein: 140 mg/dL, low-density lipoprotein: 32 mg/dL), and decreased leukocytes (white blood cells: 2.4 × 103/μL, neutrophils: 44.3%, lymphocytes: 50.9%, monocytes: 3.2%, basophils: 1.6%, eosinophils: 0%) and platelets (83 × 103/μL). However, there was no increase in the inflammatory markers. Chest X-ray showed no cardiac enlargement; however, an electrocardiogram (ECG) showed a QS pattern from V1 to 4 (Figure 1A). Echocardiography showed an ejection fraction (EF) of 8.6% and fractional shortening (FS) of 3.7%, and as wall motion; the cardiac base was distended and only the apex weakly contracted (Figure 2A). Additional blood tests were performed and showed creatine kinase (CK), CK-MB, and troponin T levels of 199 U/L, 10 U/L, and 0.022 ng/mL, respectively, with no elevation of myocardial markers. However, B-type natriuretic peptide was elevated at 144 pg/mL. The cardiologist re-evaluated the echocardiogram and made a diagnosis of basal-type TTS. Other examinations, including computed tomography of the whole body and magnetic resonance imaging of the head, revealed no obvious abnormalities. Based on the above examinations, the patient was managed in the ICU owing to impaired consciousness caused by TTS-induced hypoglycemic coma and shock.

From the time of admission, the patient was treated with hypertensive drugs to assist circulation for hypotension and intravenous glucose loading for hypoglycemia. The three hypertensive drugs used were noradrenaline 0.2 μg/kg/min, vasopressin 1.0 units/h, and dobutamine 3.0 μg/kg/min. On the second day of admission, blood tests showed mildly elevated myocardial markers: CK, 301 U/L; CK-MB, 17 U/L; and troponin T, 0.142 ng/mL. Furthermore, leads V5 and V6 on ECG showed a QS pattern, with V2-V5 and V6 also showing a QS pattern; mild ST-segment elevation was seen in V2-V6 (Figure 1B). Echocardiography on the 10th day of hospitalization showed an overall improvement in left ventricular motion, with an EF/FS of 27.9%/12.5% (Figure 2B). High-dose hypertensive drugs (noradrenaline 0.3 μg/kg/min, vasopressin 1.5 units/h, dobutamine 10 μg/kg/min) were used after the ICU admission. Her blood pressure began to increase on the seventh day of admission; all catecholamines were discontinued on the ninth day of admission. Regarding hypoglycemia, 500 mL of lactate Ringer's solution containing 25 g of glucose was administered intravenously once a day on the first two days of hospitalization, and tube feeding commenced on the third day and continued thereafter, without hypoglycemia or refeeding syndrome, hypophosphatemia, hypokalemia, hypocalcemia, hypomagnesemia, and congestive heart failure. The patient's general condition stabilized after the 10th day of hospitalization. She was discharged from the ICU on the 15th day of hospitalization. After being transferred to a general ward, the patient underwent nutritional management and rehabilitation and was discharged three months later.

The Tohoku Medical and Pharmaceutical University Ethics Committee approved this study (approval number: 2022-4-029). The patient and his family provided written informed consent for the publication of this case report and accompanying images.

## Discussion

To our knowledge, this is the first case of recurrent TTS due to hypoglycemia; TTS is a rare complication that can occur during hypoglycemia. TTS is characterized by transient ventricular dysfunction with various wall motion abnormalities and was first reported by Sato et al. in 1990 [7]. It is also known as "broken heart syndrome," "stress cardiomyopathy," or "apical ballooning syndrome" [1]. Although the pathogenesis of TTS has not yet been elucidated, a possible mechanism is a transient damage to cardiomyocytes, the substrate of TTS, due to catecholamine secretion via norepinephrine secreted from cardiac sympathetic nerves, and norepinephrine and epinephrine secreted from the adrenal medulla [8]. TTS is generally predominant in women (approximately 90%), with the average age of onset being approximately 70 years, making it more common in postmenopausal women [1]. It is highly prevalent in those with psychiatric and neurological disorders; 42.3% of patients with TTS patients are diagnosed with psychiatric disorders and often treated with antidepressants, selective serotonin reuptake inhibitors, and benzodiazepines [9]. Our patient had an eating disorder and was taking mirtazapine and clonazepam.

Triggers for the onset of TTS include psychological stress, physical stress, or both. Psychological stress includes grief, anxiety, fear, anger, frustration, interpersonal stress, and money and employment problems. Physical stress includes acute respiratory failure, postoperative issues, fractures, central nervous system disorders, infection, and malignancy [1]. TTS rarely recurs. Kato et al. reported TTS recurrence in 66 of 1402 patients (4.7%), and Christopher et al. reported TTS recurrence in 39 of 519 patients (7.5%) [10,11]. Studies that mentioned the recurrence cause found that the stress triggering TTS the second time is often the same type of stress as the first time [10,12]. Hypoglycemia has been reported as a trigger for TTS in a small number of cases, mostly in those with anorexia nervosa [5,12-14]. However, sympathetic activity in patients with anorexia nervosa is lower than that in normal subjects, which makes them less prone to TTS [14]. The mechanism of TTS due to hypoglycemia in anorexia nervosa has been described as follows. Plasma catecholamines and their metabolites decrease in anorexia nervosa, and hypoglycemia causes an increase in catecholamines [13]. The excess catecholamines stimulate β2 adrenergic receptors and shift them to inhibitory signals, causing cardiac stunting and triggering TTS [15]. Another mechanism is the "perfusion-metabolism mismatch," in which glucose uptake from the apical and middle midgut is selectively decreased during TTS, and the uptake is further reduced during hypoglycemia [16,17]. The above mechanisms are relevant and may be related to the neurogenic effects of TTS.

These mechanisms may be related to the development of TTS in patients with hypoglycemia due to anorexia nervosa. In our case, no physical stress such as acute respiratory failure, central nervous system disorder, infection, or malignancy was found, and hypoglycemia was the most obvious physical stress causing impaired consciousness. Hence, hypoglycemia with anorexia nervosa was considered a trigger for TTS. It is also important to note that vital signs are the key to considering TTS during hypoglycemia. As mentioned above, hypoglycemia is not typically associated with hypotension because it results in strong stimulation of catecholamine secretion in the adrenal medulla, hemodynamically resulting in an increased heart rate and systolic pressure and widening of pulse pressure [18,19]. However, TTS has been shown to cause cardiogenic shock in 12.2% of affected patients [9]. Thus even hypoglycemia can be associated with shock-related vital signs if TTS is present.

## Conclusions

Recurrent TTS due to hypoglycemia may develop in patients with anorexia nervosa. Anorexia nervosa is associated with decreased catecholamine secretion. An increase in catecholamine secretion due to hypoglycemia can cause cardiac stunting, leading to the development of TTS. TTS should be considered in cases of hypoglycemia with recurrent shock-related vital signs.

## References

[1] Ghadri JR, Wittstein IS, Prasad A (2018). International expert consensus document on Takotsubo syndrome (Part I): clinical characteristics, diagnostic criteria, and pathophysiology. Eur Heart J.

[2] Saito Y (2005). Hypoglycemic attack: a rare triggering factor for takotsubo cardiomyopathy. Intern Med.

[3] Kikuchi K, Yasui-Furukori N, Hasegawa C, Watahiki M, Inoue T, Shimoda K (2021). Takotsubo cardiomyopathy after hypoglycemia in a patient with anorexia nervosa. Ann Gen Psychiatry.

[4] Katoh S, Yamada Y, Shinohe R, Aoki K, Abe M (2012). Takotsubo cardiomyopathy associated with hypoglycemia: inverted takotsubo contractile pattern. Am J Emerg Med.

[5] Shimizu K, Ogura H, Wasa M, Hirose T, Shimazu T, Nagasaka H, Hirano K (2014). Refractory hypoglycemia and subsequent cardiogenic shock in starvation and refeeding: report of three cases. Nutrition.

[6] Kirigaya J, Iwahashi N, Tanaka R, Inayama Y, Takeuchi I (2022). A fatal case of takotsubo cardiomyopathy secondary to refractory hypoglycemia in severe starvation: an autopsy case report. Cureus.

[7] Sato H, Tateishi H, Uchida T (1990). Tako-tsubo-like left ventricular dysfunction due to multivessel coronary spasm. Clinical Aspect of Myocardial Injury: From Ischemia to Heart Failure (Article in Japanese).

[8] Madias JE (2021). Insulin and takotsubo syndrome: plausible pathophysiologic, diagnostic, prognostic, and therapeutic roles. Acta Diabetol.

[9] Templin C, Ghadri JR, Diekmann J (2015). Clinical features and outcomes of takotsubo (stress) cardiomyopathy. N Engl J Med.

[10] Kato K, Di Vece D, Cammann VL (2019). Takotsubo recurrence: morphological types and triggers and identification of risk factors. J Am Coll Cardiol.

[11] Lau C, Chiu S, Nayak R, Lin B, Lee MS (2021). Survival and risk of recurrence of takotsubo syndrome. Heart.

[12] Sharkey SW, Windenburg DC, Lesser JR (2010). Natural history and expansive clinical profile of stress (tako-tsubo) cardiomyopathy. J Am Coll Cardiol.

[13] Ohwada R, Hotta M, Kimura H, Takagi S, Matsuda N, Nomura K, Takano K (2005). Ampulla cardiomyopathy after hypoglycemia in three young female patients with anorexia nervosa. Intern Med.

[14] Elikowski W, Małek-Elikowska M, Marcinkowski P, Komendzińska-Ognik D, Rzymski S (2018). Takotsubo cardiomyopathy triggered by profound hypoglycemia in a 39-year-old female with anorexia nervosa: strain monitoring of left ventricle function recovery. Pol Merkur Lekarski.

[15] Paur H, Wright PT, Sikkel MB (2012). High levels of circulating epinephrine trigger apical cardiodepression in a β2-adrenergic receptor/Gi-dependent manner: a new model of Takotsubo cardiomyopathy. Circulation.

[16] Ansari MJ, Prasad A, Pellikka PA, Klarich KW (2011). Takotsubo cardiomyopathy caused by hypoglycemia: a unique association with coronary arterial calcification. Int J Cardiol.

[17] Gupta S, Goyal P, Idrees S, Aggarwal S, Bajaj D, Mattana J (2018). Association of endocrine conditions with takotsubo cardiomyopathy: a comprehensive review. J Am Heart Assoc.

[18] Towler DA, Havlin CE, Craft S, Cryer P (1993). Mechanism of awareness of hypoglycemia. Perception of neurogenic (predominantly cholinergic) rather than neuroglycopenic symptoms. Diabetes.

[19] DeRosa MA, Cryer PE (2004). Hypoglycemia and the sympathoadrenal system: neurogenic symptoms are largely the result of sympathetic neural, rather than adrenomedullary, activation. Am J Physiol Endocrinol Metab.

